# A Comprehensive Correlation of Clinicopathologic Prognostic Factors in Malignant Adult Renal Tumors: A Single Institutional Study

**DOI:** 10.5146/tjpath.2023.12849

**Published:** 2024-01-22

**Authors:** Sobiya M Ayesha, Swetha Sivakumar, Rahul Devraj, Rajsekhar Shantappa, Ranganath Ratnagiri, Meher Lakshmi Konatam, Shantveer G Uppin, Tara Roshni Paul, Megha S Uppin

**Affiliations:** Pathology, Nizams Institute of Medical Sciences, Telangana, India; Urology, Nizams Institute of Medical Sciences, Telangana, India; Surgical Oncology, Nizams Institute of Medical Sciences, Telangana, India; Medical Oncology, Nizams Institute of Medical Sciences, Telangana, India

**Keywords:** Renal cell carcinoma, Kidney neoplasms, Adult renal tumors, Prognosis, Survival

## Abstract

***Objective: *** To study the clinicopathologic prognostic parameters of malignant adult renal tumors as these have poor over-all survival (OS) and show frequent metastasis.

***Material and Methods:*** This was a retrospective analysis of the clinical and pathologic features of malignant renal tumors in adult patients from January 2011 to December 2020. All the tumors were studied with respect to age, clinical presentation, tumor type/subtype, histologic grade (WHO/ISUP grading system), TNM stage and presence of necrosis. Correlation of histopathologic features and survival analysis was done using Kaplan-Meier survival curves and Cox-regression analysis.

***Results: *** A total of 257 cases were included in the study period including 253 renal cell tumors of which clear cell renal cell carcinoma accounted for 69.3%. The age of the patients ranged from 20 to 87 years (median-52 years). The overall survival significantly reduced with increasing histologic grade, stage, and presence of necrosis. The comparison between the histological subtypes was not statistically significant. Univariate Cox-regression analysis found significant hazard ratio with increasing age, size, histologic grade (G4 vs G1), stage, and presence of necrosis. The correlation of OS with histological subtypes was not significant. Multivariate analysis also showed increased hazard ratio with increasing age, size, grade, and stage. However, the P-value was significant only for age.

***Conclusion:*** Clear cell renal cell carcinoma was the commonest type of adult renal tumor. Older age at presentation, larger tumor size, presence of necrosis, and higher histologic grade and stage were associated with poor prognosis in these patients.

## INTRODUCTION

The majority of the malignant adult renal tumors are the renal cell neoplasms. These account for 2-3% of the adult tumors. However, these are among the most lethal tumors with frequent metastases and poor overall survival (1). Hence many parameters have been studied to assess the prognosis among the high-risk groups. These factors include various clinical and histological elements like age, subtype, histologic grade, TNM stage, and presence of necrosis. Traditionally, Fuhrman nuclear grade was used, but the current WHO/ISUP grading system recommends the use of nucleolar grade for renal cell tumors. This study is our experience of clinicopathologic variables of malignant adult renal tumors and their correlation with survival.

## MATERIALS and METHODS

This was a retrospective study done at a tertiary care institute with established oncology and urology services. All cases of malignant tumors, diagnosed from January 2011 to June 2020 were retrieved from records. The study included 257 patients. The clinical data like age, gender, presenting complaints, and imaging findings were obtained from medical records. The histopathology slides were reviewed by two pathologists (MU and SU). The study was approved by Institutional Ethics Committee (Letter no. EC/NIMS/2700/2021, dated: 19.02.2021).

Both the gross and the microscopic features that are considered as prognostic parameters were studied in detail. These included size, histologic subtype, histologic grade, presence of necrosis, and TNM stage of the tumor. Also, the other parameters that are important for complete reporting of renal tumors including location, laterality, focality, sarcomatoid and rhabdoid change, lymphovascular invasion, and tumor extent were studied as well.

The WHO/ISUP grading system was used to assess the histologic grade in clear cell renal cell carcinoma (CCRCC) and papillary carcinoma (PRCC). Clear cell papillary renal cell tumor (CCPRCT) and multilocular cystic renal neoplasm of low malignant potential (MCRNLMP) were diagnosed in tumors with low WHO/ISUP grade only. Collecting duct carcinomas (CDCs) have inherent high-grade nuclei and an aggressive clinical course, which obviate the use of grading. Chromophobe RCC (ChRCC) was not graded as the WHO/ISUP system is not applicable to this subtype (2,3).

The pathological changes in the adjacent non-neoplastic renal parenchyma were also studied.

Immunohistochemistry (IHC) results were reviewed wherever performed. IHC was performed on a fully automated immunostainer (Xmatrix; Biogenex,California, USA) by the poly HRP technique. The primary antibody panel used was determined by initial morphological impression. The common panel of markers used for renal cell tumors included panCK, CK7, PAX8, CD10, AMACR, CD117, and vimentin.

### Statistical Analyis

Continuous numerical data (e.g., age, size) were studied using the median. Nominal data (e.g., gender) was analyzed by ratios. Percentages were used for both nominal and ordinal data (e.g., various clinical, gross and microscopic features including necrosis). Kaplan-Meier survival analysis was done to compare time to death between various prognostically significant groups and to know the 5-year survival estimate for each group. Cox-regression analysis (univariate and multivariate) was done to know the association of survival time with covariates as well as to calculate the hazard ratios for each variable. These were done using Statistical Package for the Social Sciences (SPSS) software version 16.

### Limitation

The retrospective nature of the study and absence of molecular work up are some of the limitations of the study.

## RESULTS

This study included a total of 257 patients who underwent nephrectomy for malignant renal tumors including 222 radical and 35 partial nephrectomies. All the tumors except two were proven to be non-metastatic on imaging at the time of surgery. These two cases had lung metastasis on PET-CT at presentation. These cases were treated with upfront surgery to reduce the tumor burden (the tumor was invading the renal vein in one and the colon in the other case), followed by adjuvant chemotherapy.

Histologically, these were divided into renal cell tumors (n=253, 98.4%) and other types (n=4, 1.6%). The most common histologic subtype was CCRCC (n=178, 69.3%), followed by PRCC (n=47, 18.2%), ChRCC (n=18, 7%), CDC (n=4, 1.6%), MCRNLMP (n=4, 1.6%), CCPRCT (n=1, 0.4%), and mucinous tubular and spindle cell carcinoma (MTSCC, n=1, 0.4%). The other four were mesenchymal tumors like synovial sarcoma, epithelioid angiomyolipoma, renal gastrointestinal stromal tumor (GIST), and neuroendocrine tumor, grade 2.

### Clinical Features

The age ranged from 20 to 87 years (median-52) including 177 male and 80 female patients. The details of the clinical and histopathologic parameters in all the tumor subtypes have been enlisted in Table 1. The most common presenting complaint was flank pain (n=105), followed by hematuria (n=58) and mass per abdomen (n=55). The classic triad of flank pain, hematuria, and abdominal mass were seen in two cases only. Twenty-two cases were detected incidentally on imaging performed for other reasons, which included CCRCC (14), PRCC (5), ChRCC (1), MCRNLMP (1) and CCPRCT (1). The single case of MTSCC was a 45-year-old female who presented with hematuria and the single case of CCPRCT was a 57-year-old male.

**Table 1:** Clinical, gross and microscopic features of renal cell tumors.

	**CCRCC**	**PRCC**	**ChRCC**	**CDC**	**MCRNLMP**
Total cases	178	47	18	4	4
Age [median]	20-80 [53]	28-87 [52]	35-75 [50]	45-65 [57]	23-61 [33]
Male: Female	2:1	3.3:1	1.6:1	4=0	4=0
**Procedure** Radical Partial	156 (87.6) 22 (12.4)	39 (83) 8 (17)	16 (88.9) 2 (11.1)	4 (100) -	1 (25) 3 (75)
**Location** Upper pole Mid pole Lower pole Entire kidney Unknown	69 (38.8) 31 (17.4) 49 (27.5) 16 (9) 13 (7.3)	14 (29.8) 9 (19.1) 14 (29.8) 7 (14.9) 3 (6.4)	7 (38.9) 3 (16.7) 5 (27.7) 3 (16.7) -	2 (50) 1 (25) 1 (25) - -	3 (75) - 1 (25) - -
**Laterality** Right Left	93 (52.2) 85 (47.8)	27 (57.4) 20 (42.6)	9 (50) 9 (50)	- 4 (100)	2 (50) 2 (50)
Size in cm [median]	1.8-24 [6.5]	2-20 [8]	2-16 [7.75]	5.3-8.5 [7.75]	4-9.5 [6.75]
Sarcomatoid change	7 (6.2)	-	1 (5.5)	1 (25)	-
Rhabdoid change	1 (0.56)	-	-	-	-
**Histologic grade** G1 G2 G3 G4	33 (18.5) 96 (54) 37 (20.8) 12 (6.7)	10 (21.3) 20 (42.6) 16 (34) 1 (2.1)	- - - -	- - - -	4 (100) - - -
Necrosis (% area)	94 (5-90)	37 (10-65)	14 (5-15)	4 (15-40)	-
LV invasion	17 (9.6)	-	-	2 (50)	-
**Invasion into:** Renal sinus fat Pelvicalyceal system Perirenal fat Renal vein IVC Gerota’s fascia Adrenal gland Adjacent colon	14 (7.8) 1 (0.55) 13 (7.3) 25 (14) 2 (1.12) 4 (2.2) 1 (0.55) 2 (1.12)	2 (4.3) - 5 (10.6) 4 (8.5) - - - -	1 (5.6) - 1 (5.6) 2 (11.1) - 1 (5.6) 1 (5.6) -	4 (100) 1 1 2 - - - -	- - - - - - - -

() brackets show percentage. **RCC:** Renal cell carcinoma, **CCRCC:** Clear cell RCC, **PRCC:** Papillary RCC, **ChRCC:** Chromophobe RCC, **CDC:** Collecting duct carcinoma, **MCRNLMP:** Multilocular cystic renal neoplasm of low malignant potential, **LV:** Lymphovascular, **IVC:** Inferior vena cava.

**Figure 1:**
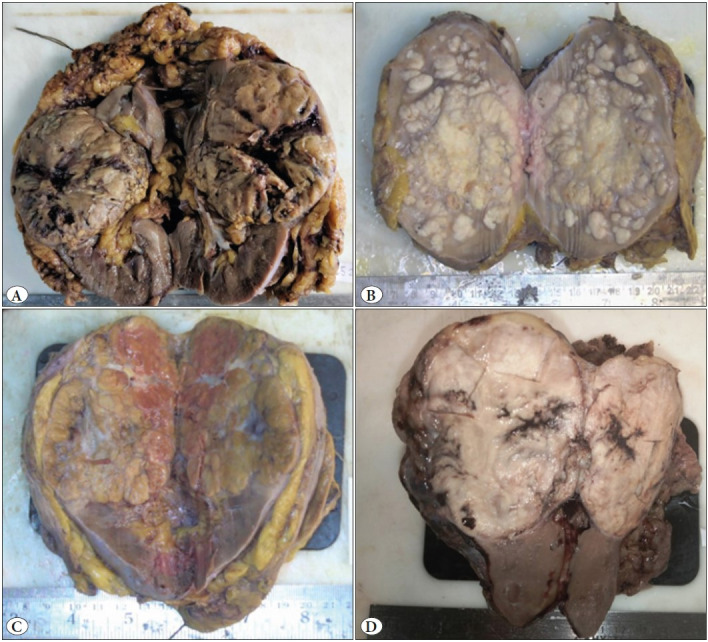
Gross images of Clear cell RCC (**A**), Papillary RCC (**B**), Chromophobe RCC (**C**) and Mucinous tubular and spindle cell carcinoma (**D**).

**Figure 2:**
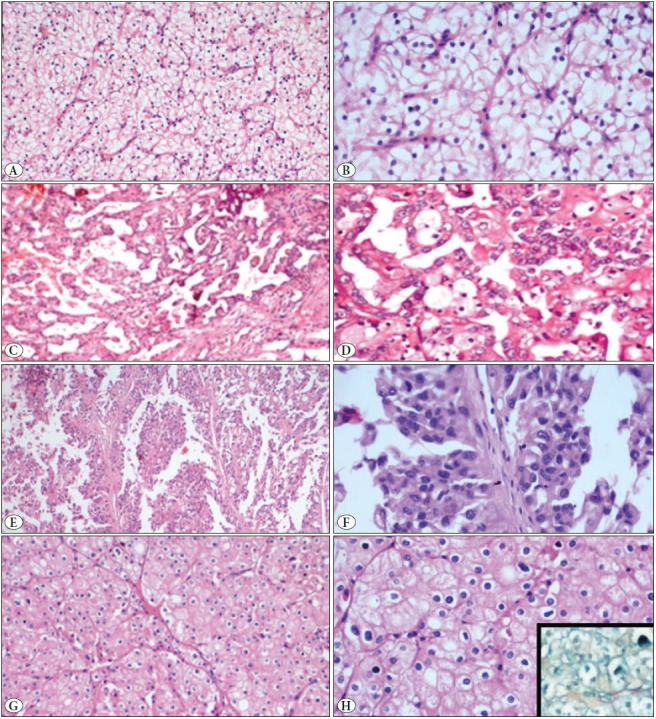
Photomicrographs showing Clear cell RCC (**A,B**), Papillary RCC (type1-**C,D**, type2- **E,F**), Chromophobe RCC (**G, H**, Inset- Hale’s colloidal iron stain, 400x). H&E stain. A,C,E,G- 200x ; B,D,F,H- 400x magnification.

The gross and microscopic features of the renal cell tumors are listed in Table 1, Figure 1 and Figure 2.

All the tumors were unifocal. PRCC was subdivided into type 1 (n=30) and type 2 (n=17) patterns histologically. The only MTSCC case was a female with an 8.5 cm tumor in the upper pole, limited to the kidney and without necrosis. Whereas, the CCPRCT patient had a 7cm tumor in the upper pole and showed grade 2 nucleoli on microscopy. The pathological stage classification of these cases is listed in Table 2.

**Table 2:** Pathologic stage classification of the renal cell tumors.

	**CCRCC**	**PRCC**	**ChRCC**	**MCRNLMP**	**CDC**
**Total cases**	**178**	**47**	**18**	**4**	**4**
T1a T1b T2a T2b T3a T3b T3c T4	38 (21.3) 49 (27.5) 29 (16.3) 6 (3.4) 47 (26.4) 2 (1.1) - 7 (4)	5 (10.6) 10 (21.3) 11 (23.4) 10 (21.3) 11 (23.4) - - -	1 (5.6) 4 (22.2) 6 (33.3) 3 (16.7) 2 (11.1) - - 2 (11.1)	1 (25) 1 (25) 2 (50) - - - - -	- - - - 4 (100) - - -
Nx N0 N1	147 (82.6) 22 (12.4) 9 (5)	34 (72.4) 8 (17) 5 (10.6)	17 (94.4) 1 (5.6) -	4 (100) - -	1 (25) 1 (25) 2 (50)
M1	2 (1.1)	-	-	-	-
**I**(T1N0M0) **II**(T2N0M0) **III** T1N1M0 T2N1M0 T3N0M0 T3N1M0 **IV** T4N0M0 T3NxM1 T4NxM1	87 (48.9) 31 (17.4) - 4 (2.3) 43 (24.2) 5 (2.8) 6 (3.4) 1 (0.5) 1 (0.5)	15 (31.9) 19 (40.4) - 2 (4.3) 8 (17) 3 (6.4) - - -	5 (27.8) 9 (50) - - 2 (11.1) - 2 (11.1) - -	2 (50) 2 (50) - - - - - - -	- - - - 1 (25) 3 (75) - - -

() brackets show percentage. **RCC:** Renal cell carcinoma, **CCRCC:** Clear cell RCC, **PRCC:** Papillary RCC, **ChRCC:** Chromophobe RCC, **MCRNLMP:** Multilocular cystic renal neoplasm of low malignant potential, **CDC:** Collecting duct carcinoma, **T:** Primary tumor, **N:** Regional lymph nodes, **M:** Distant metastasis.

The diagnosis was based on classic histopathologic features in the majority of the cases. Immunohistochemistry was done in 45 renal cell tumors (CCRCC=22, PRCC=15, ChRCC=3, CDC=3, CCPRCT=1 and MCRNLMP=1). All the CCRCC cases were positive for vimentin, PAX8, and CD10 whereas CK7 was positive in 3/9 cases. PRCC cases were all positive for CK7, vimentin, and AMACR, and CD10 was positive in 9/15 cases. The ChRCC cases were positive for panCK, CK7, and CD117, and were negative for CD10. The CDC cases were positive for CK7 and vimentin and were negative for CD10 and AMACR. The CCPRCT cases were positive for CK7, PAX8, CAIX (cup-like), focal positive for CD10, and negative for AMACR. The MCRNLMP case was positive for panCK and vimentin, and negative for CD10. The neuroendocrine tumor was positive for chromogranin and synaptophysin; GIST showed immunopositivity for CD117 and SMA; Synovial sarcoma showed immunoexpression of panCK, Bcl2 and TLE1; and epithelioid angiomyolipoma was positive for vimentin, CK7 and HMB45.

The adjacent non- neoplastic renal parenchyma in malignant renal tumors was un-remarkable in 157 (62.5%) cases. Features of chronic interstitial nephritis were identified in 48 (19.1%) cases. Parenchymal scarring and nonspecific vascular thickening and hyalinization were seen in 38 (15.1%) cases. Nodular glomerulosclerosis (diabetic nephropathy-Class III) and papillary adenoma were seen in two cases each. Focal segmental glomerulosclerosis, ADPKD, simple cysts and non-necrotizing granulomas were seen in one case each.

Follow up was available in 151 out of the total 257 patients. Of these, 147 were renal cell tumors and four were the other malignancies. Follow-up was not available in any of the four cases of CDC group and in the only case of MTSCC; hence survival data was not obtained and survival analysis could not be done for these groups. The median follow-up period was 35 months. Of the 147 renal cell tumors, 105 were alive and 42 had expired. Median overall survival (OS) could not be calculated because the median survival did not reach 50%. However, the estimated mean OS time was 75.4 months.

Sixteen patients had metastasis. Of these, fourteen patients developed metastasis post-surgery. The histologic subtype was CCRCC in fifteen and PRCC in one. Seven had metastases to the lung, four to the liver, two each to the bone and brain; and one had multiple metastases in the lung, liver and bone. All these patients received chemotherapy and thirteen patients had expired at the end of the study period. The patient with multiple metastases had a concurrent squamous cell carcinoma of cervix. Three other patients had additional malignancies like colon adenocarcinoma, breast cancer, and acute myeloid leukemia.

**Figure 3:**
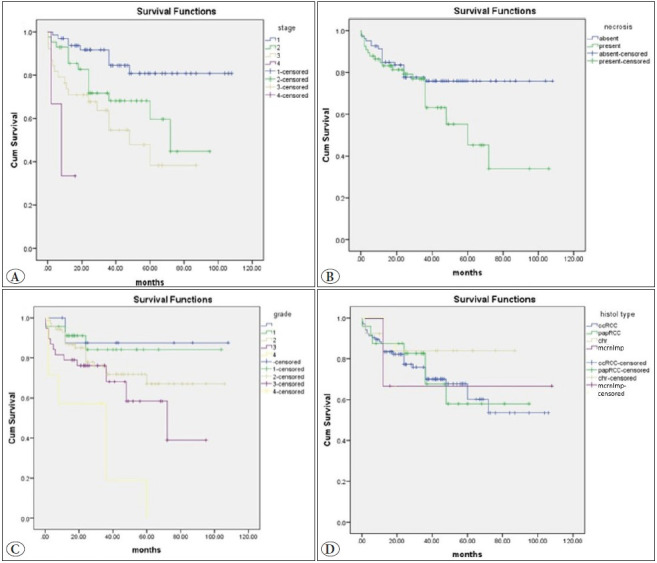
Kaplan-Meier survival graphs for (**A**) stage, (**B**) necrosis, (**C**) nuclear grade, and (**D**) histological subtypes - clear cell Renal Cell Carcinoma (ccRCC), papillary RCC (papRCC), chromophobe RCC (chr), and multilocular cystic renal neoplasm of low malignant potential (mcrnlmp).

Kaplan-Meier survival analysis was plotted and is shown in Figure 3. The OS was significantly reduced with increasing histologic grade, TNM stage, and presence of tumor necrosis. This was found to be statistically significant with corresponding p-values of 0.002, 0.0001, and 0.039 (log-rank test). However, the correlation of OS with histologic subtypes was not statistically significant (p=0.811).

The 5-year survivals for the histologic grades G1 to G4 were 84%, 67%, 58%, and 0% (G4 all expired), respectively. Similarly, for the stages I to III, it was 82%, 68%, and 47%, respectively. Of the three stage IV cases whose follow-up was possible, two patients expired (one at two months and the other at eight months post-surgery) and the third patient was alive without any disease-related complication at the time of the present study (16 months post-surgery for the third case). Hence, the stage IV patients did not reach a 5-year follow-up period and a 5-year survival could not be calculated. The 5-year survival for the patients with presence of necrosis was 45% whereas it was higher/better (76%) in those without tumor necrosis.

The 5-year survival for the histological subtypes did not show any particular trend. It was 61%, 57%, 84%, and 67% for CCRCC, PRCC, ChRCC, and MCRNLMP respectively.

Cox regression analysis was also done, as shown in Table 3. For analyzing the association between the histological subtypes and survival time, the CCPRCT group was excluded due to small sample size. Univariate analysis found significant hazard ratio with increasing age, size, histologic grade (G4 vs G1), stage, and presence of necrosis with p-value of 0.022, 0.0001, 0.008, 0.0001, and 0.045 respectively. The difference between the various histological subtypes was not significant. Only the factors which showed a statistically significant association with survival time in univariate analysis were selected for the multivariate analysis. Multivariate analysis also showed increased hazard ratio with increasing age, size, grade, and stage. However, the p-value was found to be significant only with increasing age (p-value of 0.045).

**Table 3:** Univariate and multivariate cox regression analysis of various parameters.

	**Univariate analysis**			**Multivariate analysis**		
	**HR (95% CI)**	**p**	**B**	**HR (95% CI)**	**p**	**B**
**Age**	1.032 (1.004-1.059)	0.022	0.031	1.031 (1.001-1.06)	0.045	0.031
**Size, cm**	1.150 (1.069-1.238)	0.0001	0.140	1.080 (0.975-1.19)	0.140	0.077
**Histotype (ref CCRCC)** **PRCC** **ChRCC** **MCRNLMP**	0.962 (0.389-2.063) 0.442 (0.071-1.463) 1.098 (0.061-5.160)	0.927 0.263 0.926	0.037 0.816 0.093	-	-	-
**Necrosis**	1.870 (1.014-3.450)	0.045	0.626	0.902 (0.408-1.992)	0.798	-0.104
**Grade (ref 1)**		0.008			0.269	
**Grade (2)**	2.655 (0.353-19.955)	0.343	0.976	3.494 (0.456-26.76)	0.228	1.251
**Grade (3)**	4.059 (0.532-31.001)	0.177	1.401	5.331 (0.656-43.32)	0.117	1.674
**Grade (4)**	11.557 (1.380-96.762)	0.024	2.447	8.249 (0.907-75.04)	0.061	2.110
**Stage**	2.097 (1.465-3.003)	0.0001	0.741	1.639 (1.085-2.477)	0.19	0.494

**HR:** Hazard ratio, **p:** p-value, **B:** Regression coefficient, **RCC:** Renal cell carcinoma, **CCRCC:** Clear cell RCC, **PRCC:** Papillary RCC, **ChRCC:** Chromophobe RCC, **MCRNLMP:** Multilocular cystic renal neoplasm of low malignant potential.

## DISCUSSION

The major subtypes of RCC have a differing clinical course. This study has been done to predict their outcome using various parameters like size, histologic grade, stage, and presence of necrosis.

Our study is one of the largest among the studies done in India on nephrectomies performed for malignant adult renal tumors. The median age in our study was 52 years, which is quite comparable to the other Indian studies done by Tiwari et al., Joshi et al., and Ray et al (4–6). They have also found fifth decade as the median age in their case series. This is a decade earlier than the data reported from the west (7,8). There was a male preponderance with an M:F ratio of 2.2:1, which was also observed by the other studies (4–6). This might be due to the higher prevalence of risk factors like smoking seen in the males as compared to the females (6). The commonest clinical presentation in our study was flank pain (41.5%) followed by hematuria (23%). This finding was similar to that observed by Joshi et al (5). However, hematuria was noted to be the commonest presentation by Tiwari et al. and Ray et al (4–6).

As reported in the literature, CCRCC constituted the majority (69.3%) of the malignant adult renal tumors in our study (2,4,6). The histologic grading was done according to the WHO/ISUP grading system unlike the previous studies which used the Fuhrman nuclear grading system (4,9). However, the distribution of the cases across the grades is largely similar to other studies, where G1 and G2 combined form the majority (>70%) of the cases (9). Currently, nuclear grade is integrated in many prognostic tools for renal cancer, and where applicable it is required to be reported in nephrectomies by the reporting guidelines and recommendations. It also helps in communicating the potential prognosis to the clinicians. Hence, we included the WHO/ISUP grading system along with other clinicopathologic parameters as a prognostic indicator among the adult renal cell tumors (where it is applicable) (2). However, the WHO/ISUP grading system is not validated for all histological subtypes. Alternate schemes have been proposed in literature for ChRCC, and we have not applied those as our study focused on the ‘WHO/ISUP grading system’ only. Various integrated prognostic scores have been developed, which include a few additional clinical parameters like performance status, symptoms at diagnosis, biochemical parameters like C-reactive protein, etc (10–12). However, we could not apply these prognostic scores in our study since this study focused on pathologic parameters.

In our study, the tumors in early stages (I and II) accounted for 67.5%, which was higher compared to the other studies done by Tiwari et al. and Abraham et al., where 50% or more patients presented in later stages (III and IV) (4,9). Distant metastases were observed in 6.2% (n=16) of total cases, which is lower when compared to the other Indian studies (4,5). These differences were mainly because our cases presented in the early stage as compared to the other studies. Pulmonary metastases were the commonest (50%) as expected (2). All of these patients received chemotherapy and thirteen had expired on follow-up.

Kaplan-Meier survival analysis showed that the 5-year survival was significantly reduced with increasing histologic grade and TNM stage, which is similar to other studies as shown in Table 4 (9,13,14). However, unlike the studies done by Patard et al. and Gudbjartsson et al., the comparison between the histological subtypes was not statistically significant (p=0.811) in our study (13,14). In addition, we have also observed decreasing survival with the presence of necrosis and this was found to be statistically significant.

**Table 4:** Comparison of 5-year survival with other studies.

	**Current study**		**Patard et al.[13]**		**Gudbjartsson et al.[14]**		**Abraham et al.[9]**	
	**5-yr**	**p**	**5-yr**	**p**	**5-yr**	**p**	**5-yr**	**p**
**Grade** G1 G2 G3 G4	84 67 58 0	0.002	89 72 50 28	0.0001	87 70 46 15	0.0001	91* 43┼	0.0001
**Stage** I II III IV	82 68 47 NR	0.0001	90 78 56 22	0.0001	93 80 55 11	0.0001	92╪ 64§	0.0001
**Subtype** CCRCC PRCC ChRCC MCRNLMP	61 57 84 67	0.811	64 70 84 -	0.0007	55 66 85 -	0.0001	-	-

**5-yr:** 5-year survival estimate in %, **p:** p-value, **NR:** Not reached, *: G1+G2, ┼: G3+G4, ╪: I+II, §: III+IV, **RCC:** Renal cell carcinoma, **CCRCC:** Clear cell RCC, **PRCC:** Papillary RCC, **ChRCC:** Chromophobe RCC, **MCRNLMP:** Multilocular cystic renal neoplasm of low malignant potential.

We found increasing age, size of the tumor, histologic grade, stage, and presence of necrosis to be associated with significantly decreased survival among our cases on univariate cox regression. Gudbjartsson et al., Lamb et al. and Cortellini et al. also found a similar trend in their case series with respect to age, size, grade, and stage of the tumor as depicted in Table 5 (1,14,15). There was no statistically significant difference among the various histologic subtypes. However, it is well known that the PRCC and ChRCC are related to better outcomes as compared to the CCRCC (14,16–18). This difference can be attributed to the gap between the sample size of these tumors in our study. Multivariate analysis only showed a statistically significant increase in the hazard ratio with older age at presentation.

**Table 5:** Comparison of hazard ratios with other studies.

	**Hazard Ratio (p-value)**			
	**Current study**	**Cortellini et al.[15]**	**Lamb et al.[1]**	**Gudbjartsson et al.[14]**
**Univariate analysis**				
**Age**	1.032 (0.022)	1.19 (0.4268)	1.20 (0.529)	-
**Size**	1.150 (0.0001)	-	1.01 (0.005)	1.09 (0.001)
**Histotype (ref CCRCC)** **PRCC** **ChRCC** **MCRNLMP**	0.962 (0.927) 0.442 (0.263) 1.098 (0.926)	0.58 (0.25)	-	0.60 (0.009) 0.29 (0.007) -
**Necrosis**	1.870 (0.045)	-	2.91 (0.001)	-
**Grade (4 vs 1)**	11.557 (0.024)	2.09 (0.001)	1.82 (0.001)	4.65 (0.001)
**Stage**	2.097 (0.0001)	1.65 (0.0437)	1.97 (0.001)	7.42 (0.001)
**Multivariate analysis**				
**Age**	1.031 (0.045)	-	-	1.037 (0.01)
**Size, cm**	1.080 (0.140)	-	(0.839)*	-
**Necrosis**	0.902 (0.798)	-	1.88 (0.045)	-
**Grade (4 vs 1)**	8.249 (0.061)	2.21 (0.0008)	(0.237)*	2.19 (0.03)
**Stage**	1.639 (0.19)	1.73 (0.0324)	(0.244)*	3.71 (0.001)

**RCC:** Renal cell carcinoma, **CCRCC:** Clear cell RCC, **PRCC:** Papillary RCC, **ChRCC:** Chromophobe RCC, **MCRNLMP:** Multilocular cystic renal neoplasm of low malignant potential, *= Only p-value mentioned.

The identification of non-neoplastic renal pathology in the tumor nephrectomy specimen is important as it may help in predicting the progression to declining renal function of the only kidney, which remains after nephrectomy in renal malignancies. This may also help in guiding the clinician in determining the appropriate medical management of these patients. Thirty-four percent of our cases showed non-neoplastic renal pathologies like tubulointerstitial nephritis, parenchymal scarring, and vascular change. This is lower than what was observed by Truong et al., Salvatore et al. and Bijol et al. (19–21). They found non- neoplastic pathologies in 60- 80% of their cases. This difference may be attributed to the older age of the patients in these studies as compared to our study. The median age of their cases was in the 6th or 7th decades, whereas it was 52 years in our study. Also, it is known that as the age increases, the incidence of hypertensive and diabetic nephropathy also increases, which has contributed to the non-neoplastic renal pathology in these studies.

Recently the integration of molecular and genomic factors has also been recognized as an important prognostic tool especially for targeted therapy (12). The molecularly defined renal carcinomas include TFE3-rearranged RCC, TFEB-altered RCC, ELOC-mutated RCC, FH-deficient and SDH-deficient RCC, ALK-rearranged RCC, and SMARCB1-deficient renal medullary carcinomas. Some of these are associated with characteristic morphologic features. On histology, TFE3- rearranged RCC has a papillary architecture composed of epithelioid clear cells with abundant psammoma bodies, TFEB-rearranged RCC is comprised of nests of larger epithelioid cells and smaller cells clustered around basement membrane material, ELOC-mutated RCC has a nodular appearance at low power, created by thick transecting fibromuscular bands with neoplastic cells showing voluminous clear cytoplasm and prominent cell borders, and the distinctive feature of SDH-deficient RCC is the presence of cytoplasmic vacuoles or flocculent inclusions containing eosinophilic or pale, wispy material, which may impart a bubbly appearance (2). These morphologic features were not identified in our study. Furthermore, TFE3-rearranged RCC is found to affect the pediatric population predominantly and our study concentrated on malignant adult renal tumors (22). The molecularly defined subtypes are rare and only few case reports have been described so far, in the majority of these entities (2,23).

## CONCLUSION

This study is an attempt to put forth a comprehensive association between pathology and clinical parameters of 257 malignant adult renal tumors. We found that clinical parameters like older age at presentation, and morphologic parameters like larger tumor size, presence of necrosis, higher histologic grade and TNM stage were associated with poor prognosis in these patients. The study also emphasizes detailed gross and microscopic analysis of malignant renal tumors since every parameter has a prognostic association.

## Conflict of Interest

No conflict of interest.

## Ethics Approval

The study was approved by the Institutional Ethics Committee (Letter no. EC/NIMS/2700/2021, dated: 19.02.2021).
